# Vector Competence in West African *Aedes aegypti* Is Flavivirus Species and Genotype Dependent

**DOI:** 10.1371/journal.pntd.0003153

**Published:** 2014-10-02

**Authors:** Laura B. Dickson, Irma Sanchez-Vargas, Massamba Sylla, Karen Fleming, William C. Black

**Affiliations:** Department of Microbiology, Immunology, and Pathology, Colorado State University, Fort Collins, Colorado, United States of America; University of Texas Medical Branch, United States of America

## Abstract

**Background:**

Vector competence of *Aedes aegypti* mosquitoes is a quantitative genetic trait that varies among geographic locations and among different flavivirus species and genotypes within species. The subspecies *Ae. aegypti formosus*, found mostly in sub-Saharan Africa, is considered to be refractory to both dengue (DENV) and yellow fever viruses (YFV) compared to the more globally distributed *Ae. aegypti aegypti*. Within Senegal, vector competence varies with collection site and DENV-2 viral isolate, but knowledge about the interaction of West African *Ae. aegypti* with different flaviviruses is lacking. The current study utilizes low passage isolates of dengue-2 (DENV-2-75505 sylvatic genotype) and yellow fever (YFV BA-55 -West African Genotype I, or YFV DAK 1279-West African Genotype II) from West Africa and field derived *Ae. aegypti* collected throughout Senegal to determine whether vector competence is flavivirus or virus genotype dependent.

**Methodology/Principal Findings:**

Eight collections of 20–30 mosquitoes from different sites were fed a bloodmeal containing either DENV-2 or either isolate of YFV. Midgut and disseminated infection phenotypes were determined 14 days post infection. Collections varied significantly in the rate and intensity of midgut and disseminated infection among the three viruses.

**Conclusions/Significance:**

Overall, vector competence was dependent upon both viral and vector strains. Importantly, contrary to previous studies, sylvatic collections of *Ae. aegypti* showed high levels of disseminated infection for local isolates of both DENV-2 and YFV.

## Author Summary

Vector competence is defined as the intrinsic permissiveness of an arthropod vector for infection, dissemination, and transmission of a pathogen. The mosquito *Aedes aegypti* is the main vector for dengue and yellow fever viruses worldwide and is divided into two subspecies: *Ae. aegypti aegypti* and *Ae. aegypti formosus*. *Aedes aegypti aegypti* is found globally in tropical and subtropical regions, while *Ae. aegypti formosus* is mainly restricted to sub-Saharan Africa. *Aedes aegypti formosus* is considered to be a poor vector for both yellow fever and dengue, but some of these original studies with yellow fever were performed with highly passaged viral isolates collected at different locations than the mosquitoes. Viral genetics is an important determinant of vector competence and virus/mosquito genetic specificity exists in *Ae. aegypti aegypti*. We compared the vector competence of multiple collections of *Ae. aegypti* from throughout Senegal for both yellow fever and dengue viruses to demonstrate that vector competence in *Ae. aegypti formosus* is dependent on viral genotype. In contrast to earlier claims, populations of *Ae. aegypti* in West Africa can be competent vectors of flaviviruses.

## Introduction

*Aedes aegypti* is the primary vector of yellow fever virus (YFV) and all four serotypes of dengue viruses (DENV1-4), as well as being a known vector of chikungunya virus. Dengue remains an important public health problem with an expected 390 million cases per year [1]. Although there are fewer dengue cases in Africa compared to other regions, there are 11 African countries endemic for DENV, and the World Health Organization (WHO) continually reports DENV outbreaks in previously unreported geographic areas. Furthermore, it was recently estimated that in 2007 alone, 656 million fevers occurred in African children under the age of five [2]. However, in only 78 million of these cases was it likely that the child was infected with *Plasmodium falciparum*. Despite the enormous number of acute non-malarial febrile illnesses in sub-Saharan Africa, their etiologies are poorly defined [2].

Despite an effective vaccine, YF outbreaks still occur. Cases are occasionally reported in South America (11 endemic countries), but a majority of the cases are in Africa (33 endemic countries), and most of the outbreaks are in West Africa [3]. According to the WHO 200,000 cases of YFV causes 30,000 deaths each year [4].

Both YFV and DENV belong to the family *Flaviviridae* and have a single-stranded positive sense RNA genome. There are four antigenically distinct serotypes of DENV (1-4) and all four serotypes are currently found in Africa. In West Africa, DENV-2 is an important serotype because it includes the sylvatic genotype with a high potential for emergence [5]–[7]. Sylvatic genotypes are transmitted between monkeys in forested areas, while cosmopolitan genotypes are transmitted between humans in urban areas. The sylvatic genotype has been isolated from mosquitoes, monkeys, and humans [8]–[10] in West Africa, but is genetically distinct from epidemic isolates [11], [12]. Different genotypes as well as different lineages within genotypes can result in differences in both vector capacity and the severity of human disease [13]. Genetic differences in YFV isolates are also an important predictor of vector capacity. There are seven genotypes of YFV found worldwide, two of which (West African Genotypes I and II) are endemic in West Africa. The genetic differences among YFV isolates are geographically associated with outbreaks in Africa. Specifically, West Africa genotype I is responsible for a majority of outbreaks and is genetically heterogeneous relative to other genotypes [14].

The vector for both viruses, *Aedes aegypti (L)*, exists as two subspecies: *Ae. aegypti aegypti* and *Ae. aegypti formosus* [15], [16]. Characters that distinguish the two subspecies were developed primarily in East Africa but are contradictory and confusing when identifying *Ae. aegypti* forms collected in West Africa. The current definition of the subspecies is based on the number or degree of white scales on the first abdominal tergite as defined by Mattingly and McClelland [15], [16]. *Aedes aegypti aegypti* has scales on the first abdominal tergite, has a light tan cuticle, is globally distributed, tends to be endophilic, and has a feeding preference for humans. In contrast, *Ae. aegypti formosus* has no white scales on the first abdominal tergite, has a dark or black cuticle, is found mostly in Sub-Saharan Africa in sylvatic environments, tends to be exophilic, and has a feeding preference for wild animals [17]–[19]. However, these distinctions become unclear in West Africa where *Ae. aegypti* with a dark black cuticle and white scales (albeit usually few) are frequently detected. Furthermore, *Ae. aegypti* without scales frequently breed near human habitats and bite humans. In East Africa, the scaling pattern and behavior are also correlated with discrete genetic differences in allozymes and microsatellites [20], [21]. But in West Africa, the scaling pattern does not correlate with these genetic markers [22], [23] or behavioral differences and leads to confusion in subspecies identification. Further, these genetic studies reveal that East African *Ae. aegypti aegypti* and *Ae. aegypti formosus* are genetically distinct from the monophyletic West African *Ae. aegypti* [20], [21]. Due to this ambiguity the present study will hereafter refer to all *Ae. aegypti* collections based on their breeding site, habitat, and phytogeographic region of the collection site in Senegal.

Previous vector competence studies on *Ae. aegypti* from West Africa have shown these mosquitoes to be more refractory for both DENV [24], [25] and YFV[24], [26] compared to *Ae. aegypti* collected worldwide. Studies that have specifically examined collections across Senegal showed wide variation in vector competence for both high-passage [22] and low passage field isolates of DENV-2 [27], [28]. In particular, sylvatic collections from southeastern Senegal were more refractory than other collections from throughout Senegal.

Great variation in vector competence is seen within closely related collections of *Ae. aegypti* [29]–[32] and different isolates of DENV-2 [27], [28] or YFV [33]. Furthermore, geographically distinct collections of *Ae. aegypti* from Senegal are genetically diverse [22], [23]. It has been demonstrated that vector competence of *Ae. aegypti* for DENV is governed by interactions between mosquito strain and virus genotype in natural collections [34]. This underscores the importance of using viruses and vectors that are geographically proximate and genetically diverse to draw conclusions about vector competence among collections. However, mosquito strain by flavivirus genotype interactions have yet to be examined in West African *Ae. aegypti*. Therefore, the objective of the current study was to examine these interactions by quantifying the vector competence of West African *Ae. aegypti* populations to DENV-2 and YFV field isolates.

## Methods

### Mosquito Collections

*Aedes aegypti* were collected as larvae in 8 locations in Senegal (Table 1). The sylvatic collections from the southeast (PK10 [35], and Kedougou,) were made in 2011, all others were made in 2010. The larvae were transported to a temporary local laboratory in Kedougou or Theis, reared to adults, and given a bloodmeal to generate eggs to start a colony. Eggs from each collection were brought back to Colorado State University and maintained for approximately 10–15 generations before being challenged with an artificial bloodmeal containing virus as described below. It was intended to use collections with fewer generations, but we were not able to get F_1_ mosquitoes collected from the field to survive, mate, or bloodfeed sufficiently to perform vector competence assays. Adult mosquitoes were kept in incubators maintained at 28°C with 70–80% relative humidity and a 12∶12 hour photoperiod. Eggs were collected on filter papers and stored for up to 5 months in a high humidity chamber. The collection sites consist of domestic sites around huts in urban environments and rural villages, tires in urban environments, and forests (sylvatic) where mosquito larvae were collected from treeholes and the discarded fruit husks of *Saba senegalensis* [35] (Table 1).

**Table 1:** Mosquito collections.

	Date Collected	Breeding Site	Habitat	Phytogeographic Region	Latitude	Longitude
Fatick	August 2010	Tire	Urban	Acacia-Savanna	14°20′21.5″N	16°24′38.8″W
Bignona	September 2011	Water Jar	Urban	Marsh/Swamp	12°48′6.1″N	16°13′37.8″W
Richard Toll	October 2010	Tire	Urban	Acacia-Savanna	16°27′54.1″N	15°41′1.8″W
Goudiry	October 2010	Tire	Urban	Acacia-Savanna	14°11′2.2″N	12°42′57.3″W
Kedougou	September 2011	Tire	Rural village	Deciduous Forest/Shrub	12°33′29.4″N	12°11′19.0″W
PK10	September 2011	Treehole	Forest Gallery	Deciduous Forest/Shrub	12°36′45.1″N	12°14′51.2″W
Mont Rolland	August 2010	Water Jar	Urban	Acacia-Savanna	14°55′18.1″N	16°59′37.3″W
Rufisque	October 2010	Tire	Urban	Acacia-Savanna	14°42′56.8″N	17°16′15.0″W

### Viruses

Two low passage genotypes of YFV from West Africa were used in these studies. The YFV BA-55 from Nigeria is representative of West African Genotype I and YFV DAK 1279 from Senegal is a West African Genotype II [14]. YFV BA-55 was isolated from a human during an outbreak in Nigeria [36] and has been used in previous vector competence studies [26], [33]. The DENV-2 isolate was from a sylvatic mosquito in Kedougou, Senegal (Table 2). YFV BA-55 and DAK 1279 were inoculated into Vero cells and DENV-2-75505 was inoculated into C6/36 cells to generate a virus stock. C6/36 cells were used for DENV-2-75505 to insure virus recovery because this isolate came from a mosquito and there was no data on the titer of the initial stock. The supernatants from these infections were clarified and aliquoted and stored in minimum essential media (MEM) with 20% fetal bovine serum (FBS) at −80°C for all subsequent use. Growth curves were performed on all viruses to determine the optimal number of days post-infection on which to harvest the virus for mosquito oral infection. For mosquito feeds, C6/36 cells were infected with DENV-2-75505 at a multiplicity of infection (MOI) of 0.001 or YFV BA-55 and DAK 1279 at an MOI of 0.01 and grown to a titer of approximately nine logs of plaque forming units (PFU) before being mixed with defibrinated sheep blood for a titer of approximately six logs for the mosquito feeds. The medium was removed from the C6/36 cells infected with DENV-2-75505 six days post-infection and replaced with fresh medium. Final virus was harvested 12 days post-infection and fed directly to mosquitoes. Both isolates of YFV were grown in C6/36 cells for five days before being harvested and fed directly to mosquitoes.

**Table 2:** Viral isolates.

	Isolated from	Location	Year	Passage History
DENV-2-75505	*Aedes luteocephalus*	Kedougou, Senegal	1990	AP61 p6, C6/36 p4, Vero p1
DENV-2- JAM1409	Human	Jamaica	1983	C6/36 >25 times
YFV BA-55	Human	Nigeria	1986	Suckling Mice p2, Vero p1
YFV Dak1279	Mosquito	Diourbel, Senegal	1965	Suckling Mice p6

### Mosquito Infections

Groups of approximately 30 five to seven day old mosquitoes from each colony were exposed to a bloodmeal containing approximately six to seven logs of virus for 30 minutes. The bloodmeal titer was determined from the blood before the feed. Almost all of the mosquitoes fed within 10 minutes, so the pre-feed blood represents the bloodmeal titer ingested. Females that were not completely engorged and males were removed from the study immediately following the bloodmeal. The fully engorged female mosquitoes were held for 14 days at 28°C, 70–80% relative humidity, 12∶12 hour photoperiod, and were fed water and raisins under BSL3 containment. After 14 days, legs, heads/thoraces, and midguts were separated into individual tubes. Each tissue was triturated in 100 µL (legs and midguts) or 200 µL (heads/thoraces) minimum essential medium (MEM) media with 20% fetal bovine serum and 1.5 µg/ml Fungizone. Virus titer in each sample was determined by plaque assay. The manipulation of DENV-2-JAM1409 is very similar and was previously described [37].

### Plaque Assays

All plaque assays were performed on Vero cells in 12-well tissue culture plates. When Vero cells reached 95% confluency, each triturated sample was diluted and added directly to the cells and allowed to incubate for 1 hour at 37°C. After 1 hour, the first overlay (19.6% 10× Earle's Buffered Salt Solution, 63.4% water, 6.6% Yeast extract-Lactalbumin hydrolysate, 4% fetal bovine serum, 6% sodium bicarbonate, 0.3% Gentamycin, and 0.3% Fungizone mixed 1∶1 with 2% SeaKem LE agarose) was applied. Four days post infection with either YFV isolate, or 7 days with DENV-2-75505, the second overlay (same as the first overlay plus 2.0 ml of Neutral Red per 100 ml overlay) was applied. Plaques were counted for 3 days following the addition of the second overlay. The plaque assays for DENV-2-JAM1409 were similar and have been previously described [37].

### Data Analysis

For each collection, disseminated infection (DI), midgut escape barrier (MEB) rates, and midgut infection barrier (MIB) rates were calculated. The number of mosquitoes with a DI is the number of mosquitoes with virus in the head/thorax or legs divided by the total number of bloodfed mosquitoes. The MEB rate is the number of mosquitoes without virus in the head/thorax or legs divided by the total number of midgut infected mosquitoes, and MIB is the number of mosquitoes without virus in the midgut divided by the total number of bloodfed mosquitoes. Proportions of infected mosquitoes were compared among collection sites (villages) and among viral isolates by calculating Bayesian 95% Highest Density Intervals (95% HDI) using WinBUGS 1.4 [38] and an analysis of contingency tables script (Box 6.13 in [39]). Mean virus titers were compared among isolates at each of four locations using a one way ANOVA script (Box 6.1 in [39]) run with WinBUGS and comparing 95% HDI among isolates. We used two-way ANOVA in R [model = summary(aov (Midgut∼Virus*Village))] to test for significant virus by village interactions. Correlation analyses were also performed in R [cor.test (BloodmealTiter, ProportionDisseminated)].

## Results

Vector competence was measured in 647 mosquitoes and each village-virus combination represented 14–30 mosquitoes (mean = 28 and median = 30). Due to the necessity of using freshly grown YFV [40], it was difficult to obtain identical titers in all bloodmeals. Nevertheless, bloodmeal titers were not correlated with the midgut infection (MI) rate or disseminated infection (DI) for YFV BA-55 (MI: Pearson Correlation Coefficient (r) = 0.008; DI: r = 0.18). Correlation analysis was not performed for the other three viruses because only one or two bloodmeal titers were used. In some collections, legs were used instead of the head/thorax to measure disseminated infection with YFV BA-55. But the tissue analyzed did not affect either comparisons of infection frequencies or titers. The infection frequencies (r = 0.97, P = 0.03) and titers (r = 0.87, P = 0.01) whether assaying legs or heads were strongly correlated. This correlation analysis was performed on infection data from head/thorax or legs of mosquitoes from the same collection sites infected with YFV BA-55.

### Vector Competence throughout Senegal

**Figure 1:**
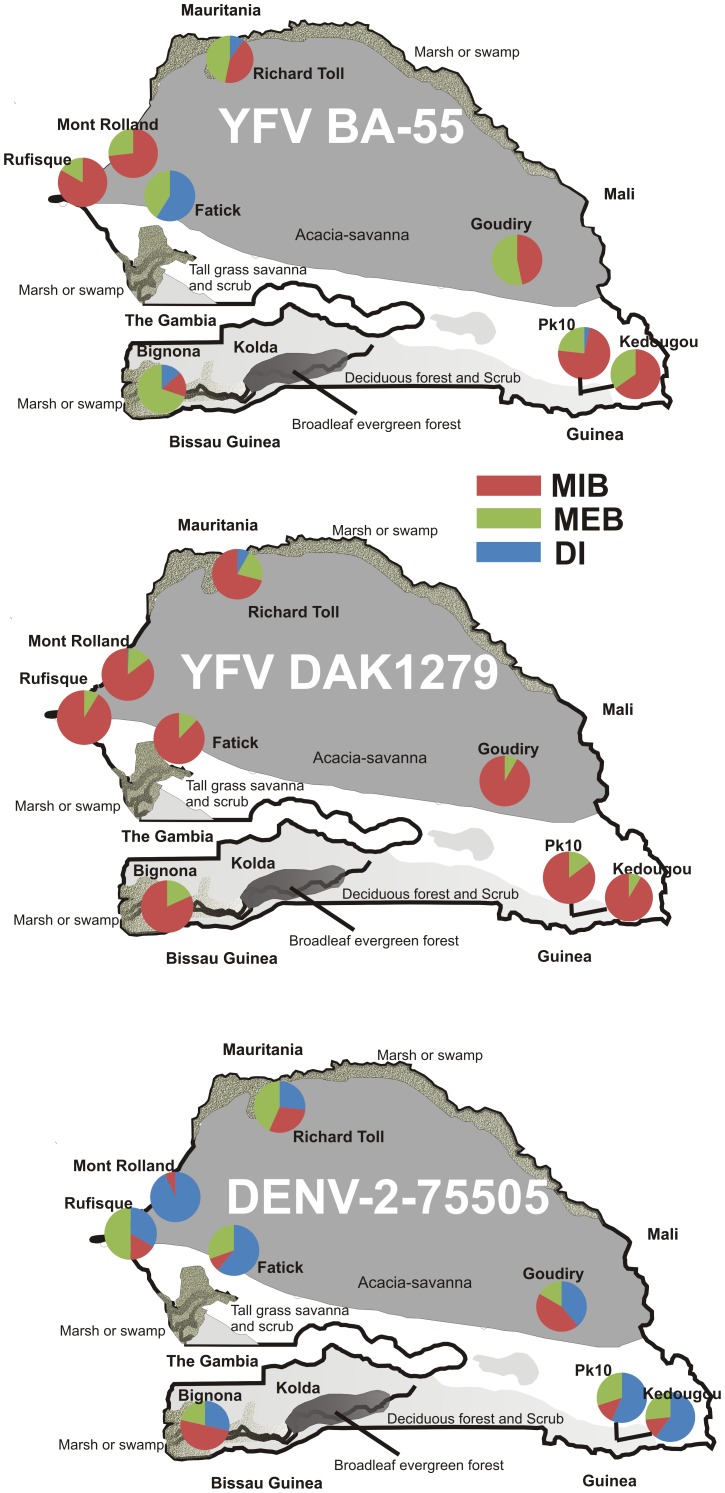
Vector Competence for DENV-2 and YFV throughout Senegal. Pie charts represent disseminated infection (DI), midgut escape barrier (MEB), and midgut infection barrier (MIB). The DI rate is the number of mosquitoes with virus in the head/thorax or legs divided by the total number of mosquitoes that bloodfed. The MEB rate is the number of mosquitoes without virus in the head/thorax or legs divided by the total number of midgut infected mosquitoes, and MIB is the number of mosquitoes without virus in the midgut divided by the total number of bloodfed mosquitoes.

Vector competence varied among collections and viral isolates (Figure 1, Table 3). The proportion and distribution of mosquitoes with a DI, MEB, and MIB infected with YFV BA-55, YFV DAK1279, or DENV-2-75505 varied across Senegal (Figure 1). Collection sites in the forested area of southeast Senegal were more refractory to YFV BA-55 than the collection sites in western Senegal with the exception of collections in urban sites close to or within the capital of Dakar. Other collections from western Senegal (Richard Toll, Fatick, and Bignona) had appreciable, albeit variable, DI rates due to a low frequency of both MIB and MEB. Mosquitoes from Fatick in particular had a 100% MI. In contrast, when infected with YFV DAK1279 the same populations of mosquitoes were highly refractory. Only five mosquitoes from Richard Toll and one mosquito from Mont Rolland developed a DI.

**Table 3:** Proportions of *Aedes aegypti* with YFV or DENV-2 infected midguts (MI) or disseminated infections (DI).

	YFV BA-55			DENV-2-75505			YFV-DAK1279		
	Titer (log_10_(PFU/ml)	MI #positive/N (%)	DI #positive/N (%)	Titer(log_10_(PFU/ml)	MI #positive/N (%)	DI #positive/N (%)	Titer(log_10_(PFU/ml)	MI #positive/N (%)	DI #positive/N (%)
**Fatick**	6.22	17/17 (100)	10/17 (59)*	6.02	21/23 (91)	14/23 (61)	5.90	3/18 (17)	0/18 (0)
**Bignona**	6.22	25/30 (83)	4/30 (13)*	6.02	7/14 (50)	4/14 (29)	7.79	10/30 (33)	0/30 (0)
**Richard Toll**	6.32	17/30 (57)	3/30 (10)*	6.02	21/30 (70)	8/30 (30)	7.79	17/30 (57)	5/30 (17)
**Goudiry**	6.04	16/30 (53)	0/30 (0)*	6.02	10/18 (56)	7/18 (39)	5.90	3/30(10)	0/30 (0)
**Kedougou**	5.34	8/23 (35)	0/20 (0)	6.02	26/30 (87)	18/30 (60)	5.90	3/29 (10)	0/29 (0)
**PK10**	6.04	8/30 (27)	1/30 (3)*	6.02	26/30 (87)	17/30 (57)	5.90	6/27 (22)	0/27 (0)
**Mont Rolland**	6.2	8/30 (27)	0/30 (0)	7.00	25/30 (83)	28/30 (93)	5.90	6/30 (20)	1/30 (3)
**Rufisque**	6.13	5/30 (17)	0/30 (0)*	6.02	25/30 (83)	10/30 (33)	5.90	3/28 (11)	0/28 (0)

Disseminated infections were assessed in either the head/thorax or the legs. Infection status was determined by plaque assay 14 days post infection.

* Disseminated infection determined using legs.

Infection rates with DENV-2-75505 were different than both isolates of YFV (Figure 1). In contrast to Sylla et al. [22], the collections from Kedougou and PK10 had a greater number of individuals with a DI when infected with DENV-2-75505 rather than DENV-2-JAM1409 as previously reported. We confirmed similar infection rates with DENV-2 JAM1409 to those reported in Sylla et al. [22] that used younger generation mosquitoes, indicating the high DI rates are not attributed to multiple generations in the lab (Table S1). The large number of individuals with a DI is especially noteworthy because the Kedougou and PK10 collection sites are sylvatic and 84% and 82% respectively of the females collected had no scales on the first abdominal tergite, thereby classifying them as classical *Ae. aegypti formosus* based on the McClelland scale [35]. Notably, all collections that were tested with DENV-2-75505 developed a disseminated infection. There was no clear association with geographic location and susceptibility. Note that the Mont Rolland and Rufisque sites in the west and Kedougou and PK10 in the southeast that were refractory to YFV BA-55 and YFV DAK1279 had a greater number of individuals with a DI when infected with DENV-2-75505.

### Mosquito/Virus Specificity

**Figure 2:**
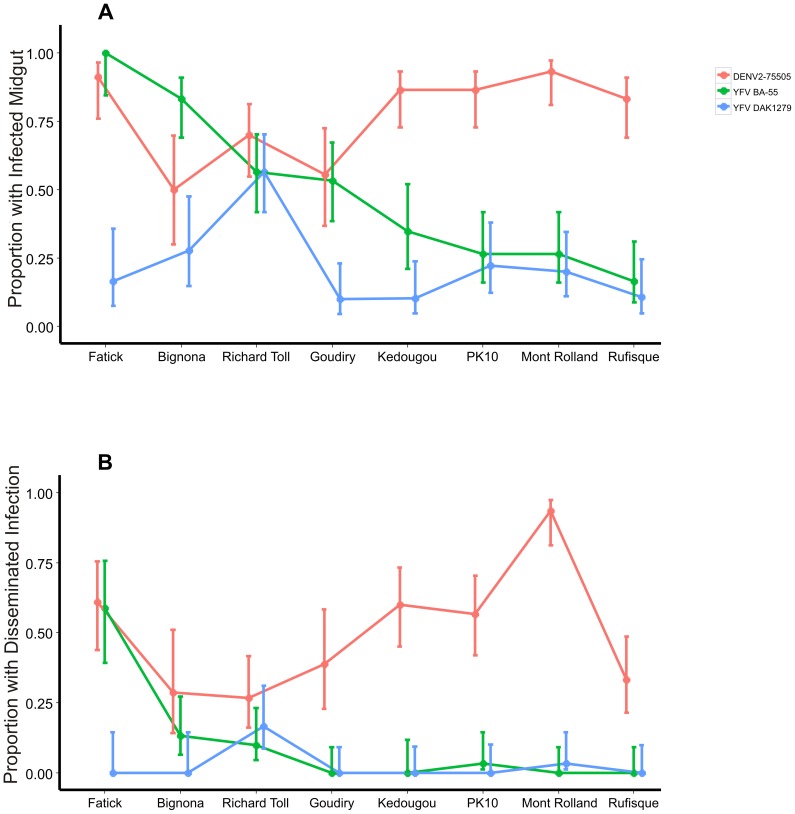
Proportion of midgut and disseminated infections. (A) The proportion of individuals with a midgut infection 14 DPI. (B) The proportion of individuals with a disseminated infection 14 DPI. Infection was determined by plaque assay. Error bars represent Bayesian 95% credible intervals and non-overlapping error bars indicate statistical significance.

To test for a mosquito strain by virus genotype interaction, the proportion of MIs and DIs were compared among the eight geographically distinct collection sites and three viruses. The proportion of MIs and DIs was dependent on both the virus and collection site (Figures 2a and 2b), indicating a mosquito strain by virus genotype interaction. Significant differences in the proportion of MIs and DIs were seen in all the collection sites, except for Richard Toll. There was no significant difference in MI rate in mosquitoes from Goudiry infected with DENV-2 75505 or YFV BA-55, but the DI rate was significantly different. Also, MI rates in Bignona were significantly different between YFV BA-55 and YFV DAK1279, but DI rates were not significantly different (Figures 2a and 2b).

**Figure 3:**
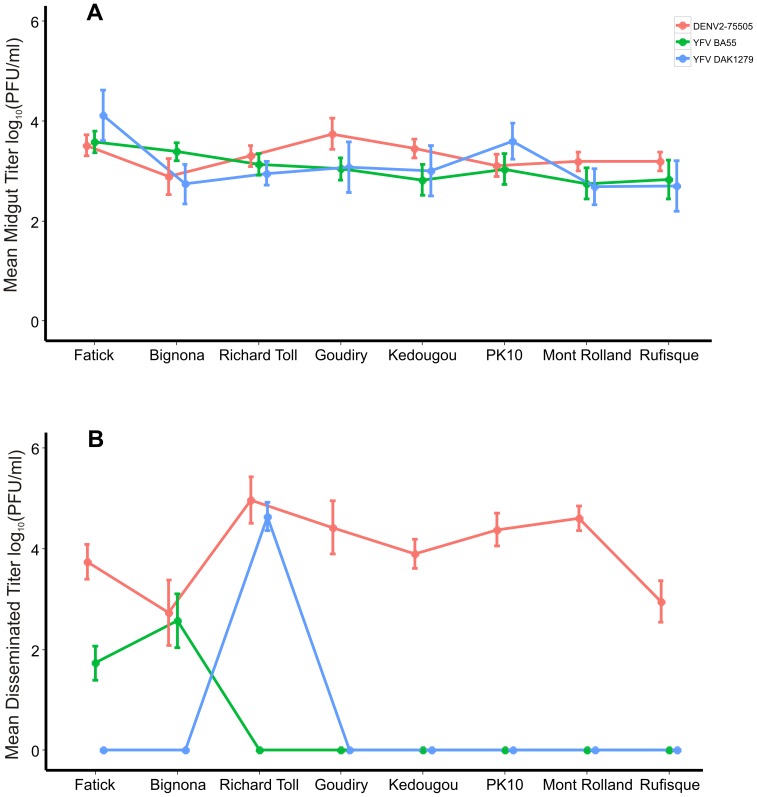
Mean virus titer in midgut and disseminated infections. (A) The mean viral titer in the midgut. (B) The mean viral titer in disseminated infections. Virus titers were determined 14 DPI by plaque assay. Error bars represent Bayesian 95% credible intervals and non-overlapping error bars indicate statistical significance.

**Figure 4:**
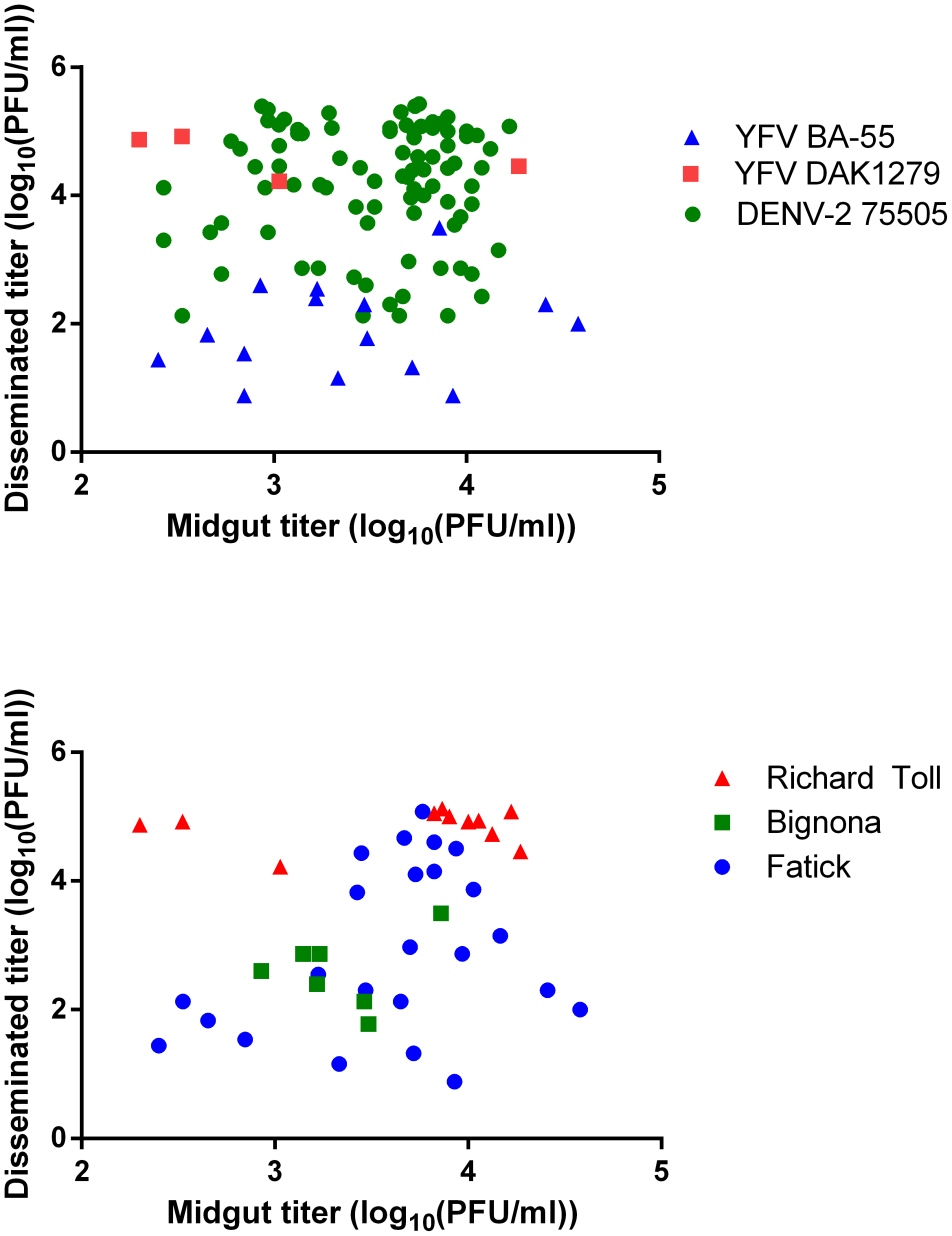
The titer of virus in the midgut is not correlated with the titer of virus in other tissues. Virus titer in midguts (log_10_(PFU/ml)) was compared with virus titer in DIs (log_10_(PFU/ml)) from the same individual. The analyses were grouped by viral isolate (A) or collection site (B). The collection sites shown in (B) are the only 3 that had individuals with a disseminated infection with all 3 viruses and each point represents an individual. Pearson correlation coefficients: DENV-2-75505 (r = 0.041, P = 0.707), YFV DAK1279 (r = −0.607, P = 0.394), YFV BA-55 (r = 0.213, P = 0.446), Fatick (r = 0.309, P = 0.143), Richard Toll (r = 0.156, P = 0.647), Bignona (r = 0.231, P = 0.619).

Although, the MI rate was variable among collections and viruses, the titer of virus in mosquitoes that developed a MI were similar among the eight collection sites and the three viruses (Figure 3a). The average titer of virus in the midgut was distributed around 3 logs (Figure 3a) and ranged from 2.5–4.5 logs (Figure 4) in individual mosquitoes. In contrast there was a broad distribution of viral titers in DIs (Figure 3b) extending from zero up to 5.5 logs and ranged from 1.5–5.5 logs (Figure 4). The titer of virus was dependent on the virus, the collection site, and the virus by collection site interaction in the midgut (F-statistic = 3.41; P - value = 1.79×10^−2^) and in DI (F-statistic = 28.50; P - value = 4.91×10^−4^) (Figure 3).

The titer of virus in MIs was not correlated with the titer of virus in DIs regardless of viral isolate (Figure 4a) or collection site (Figure 4b). Only three collection sites (Richard Toll, Bignona, and Fatick) had individuals that developed a DI with all three viruses (Figure 4b). In general, as reported earlier [25], [26] the efficiency of viral replication in the midgut does not affect the efficiency of viral replication in other tissues during dissemination.

**Figure 5:**
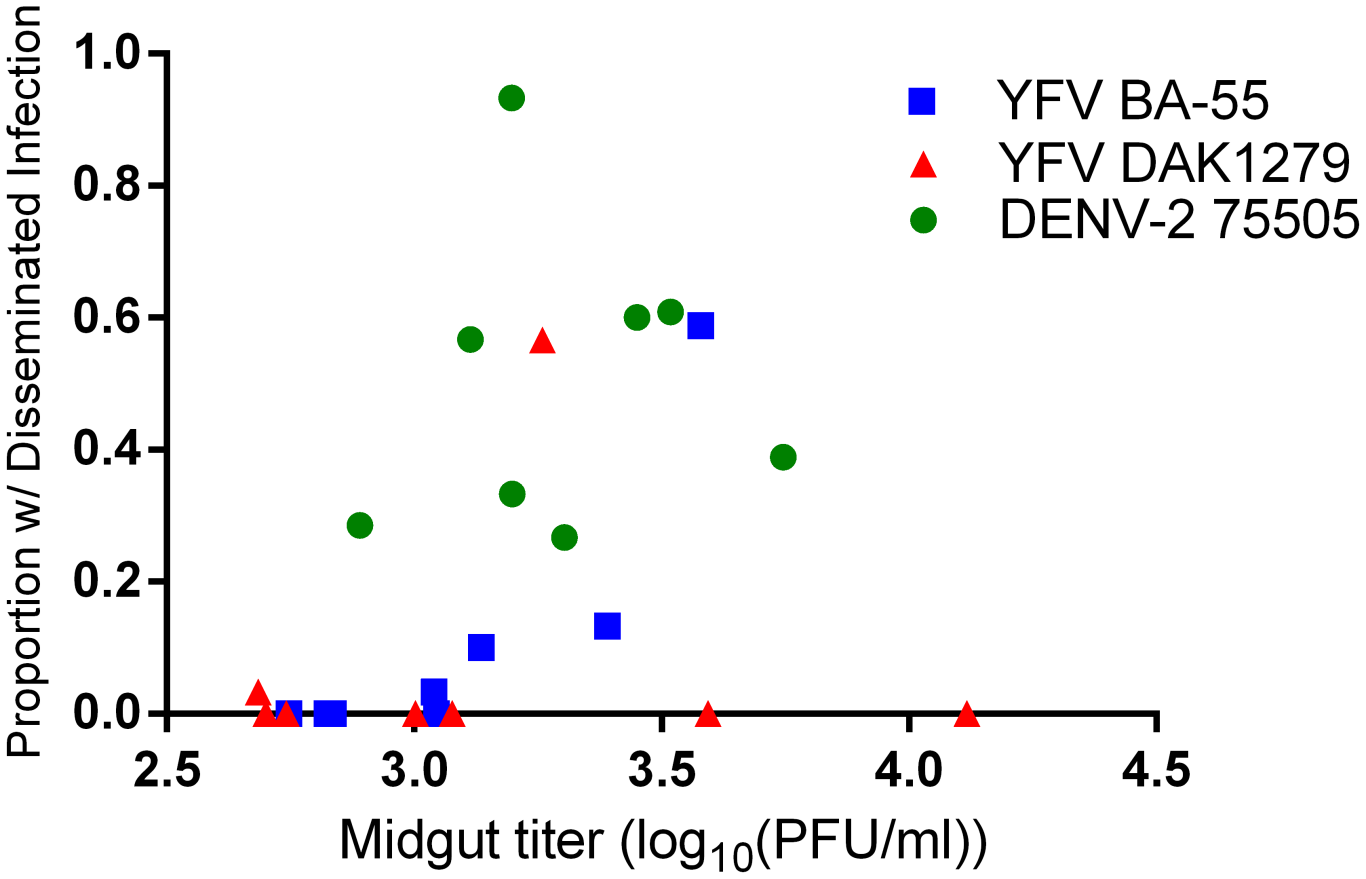
The titer of virus in the midgut is correlated with dissemination rate upon infection with YFV BA-55. Mean virus titers (log_10_(PFU/ml)) in the midgut for each of the 8 collection sites were compared with the proportion of individuals in that collection that developed a disseminated infection using a Pearson correlation analysis. Mean viral titer in the midgut was correlated with the proportion of individuals that developed a DI upon infection with YFV BA-55(r = 0.843, P = 0.0086). There was no correlation in YFV DAK279 or DENV-2-75505.

The mean titer of virus in MIs was correlated with the proportion of individuals within a collection site that developed DIs with YFV BA-55 (r = 0.843; P = 0.0086), but not with YFV DAK1279 or DENV-2-75505 (Figure 5). No correlation existed between midgut titer and the proportion of individuals with a DI when analyzed by collection site. This indicates that MEB and DI rates are independent of the efficiency of viral replication in the midgut, but in a virus dependent manner.

## Discussion

We document great variability in vector competence for both DENV-2 and YFV in collections of *Ae. aegypti* from across Senegal. The northwest-southeast decline in the susceptibility to YFV BA-55 is very similar to that seen with DENV-2-JAM1409 [22]. However, contrary to previous work with DENV-2 JAM-1409, the same collections from across Senegal, including sylvatic collections, developed a DI with DENV-2-75505, an isolate of DENV-2 from the same region. Comparison of infection rates and the titer of virus revealed a mosquito strain by virus genotype interaction during MI and DI. Although there was significant variability in MI rates, the titer of virus in the midgut was similar among viruses and mosquito collections. The titer of virus in DIs was much more variable among viruses and mosquito collections than MI titers. The efficiency of viral replication in the midgut was not correlated with the efficiency of viral replication in other tissues during DI. The proportion of individuals in each collection that developed a DI was correlated with the efficiency of YFV BA-55 viral replication in the midgut. In contrast, the proportion of individuals in each collection that developed a DI was not correlated with the efficiency of YFV DAK1279 or DENV-2-75505 viral replication in the midgut The current study builds on previous work by quantifying differences in infection rates and viral titers between two field isolates of YFV (YFV BA-55 and YFV DAK 1279) and a field isolate of DENV-2 (DENV-2-75505) in the same mosquito stains with known genetic diversity [22], [23].

Previous studies on the vector competence of West African *Ae. aegypti* for YFV [24], [26] suggested that West African *Ae. aegypti* are more refractory to YFV infection than *Ae. aegypti* from the Americas and Asia. Tabachnick et al. [24] showed that two *Ae. aegypti* collections from western Senegal when infected with the Asibi isolate of YFV, were more refractory than collections from the Americas or Asia. Although the Asibi isolate is from Ghana, it had been passaged many times and may not have been representative of isolates involved in natural transmission cycles. Similar to their western Senegal collections, collections close to Dakar were more refractory than other collections throughout the country. Miller and Mitchell [26] showed that an *Ae. aegypti* collection from Nigeria was much more refractory (10% DI) when compared with collections from the Americas (90% DI) to a YFV isolate from Peru and was completely refractory (0% DI) with YFV BA-55, the same isolate used in the present study. The current study demonstrates that some collections from Senegal also had 0% DI when infected with YFV BA-55, but that other collections did develop DIs. Although it is difficult to draw an informative conclusion by comparing results obtained with mosquitoes from Nigeria with the results obtained here, the discrepancy in vector competence between the two populations with the same viral isolate further demonstrates specificity of the virus/mosquito interaction for vector competence.

A number of studies of *Ae. aegypti* from Senegal have examined vector competence for DENV-2 [22], [24], [25], [27], [28] Those results differ from those presented here. When compared with *Ae. aegypti* from the Americas or Asia, West African *Ae. aegypti* were less susceptible to DENV-2 [24], [25]. In studies directly comparing collections within Senegal [22], [27], [28], there was variation in susceptibility, but the sylvatic *Ae. aegypti* were more refractory than domestic *Ae. aegypti*. However, the study by Sylla et al. [22] only examined the highly passaged DENV-2-Jam1409 isolate. Measuring vector competence in *Ae. aegypti* with a viral isolate collected in proximity may be the most informative approach [34]. Diallo et al. [27], [28] did so by examining the vector competence of *Ae. aegypti* from Senegal with multiple local isolates of DENV-2. Diallo et al. [28] reported that sylvatic *Ae. aegypti* collections have lower infection rates than other sylvatic species of *Aedes*, but some sylvatic *Ae. aegypti* mosquitos developed a DI. Diallo et al. [27] reported low levels of midgut infection (0.0–26.3%) and variable disseminated infection (0–100%) in six collections from Senegal regardless of geographic location. Importantly, both studies demonstrated variability in infection rates based on the isolate of DENV-2 and the collection site.

The high rates of infection with DENV-2-75505 in sylvatic collections in the current study are not congruent with the low rates of infection in the previous studies [22], [27], [28]. An explanation for our differing results could be a result of multiple generations in the lab. Unlike Diallo et al. [27], we were unable to get F_1_ mosquitoes collected from the field to survive, mate, or bloodfeed sufficiently to perform vector competence assays. High DI rates as a result of lab adaptation are unlikely because similar DI rates with DENV-2-JAM1409 confirmed that the older generation mosquito collections used in this study had similar vector competence as our younger generation mosquitoes previously reported [22]. Another explanation could be that the DENV-2-75505 isolate is more infectious in these mosquito populations than isolates used by Diallo et al. [27], highlighting the importance of the viral isolate in vector competence assays.

These populations were not screened for insect only viruses, therefore we cannot rule out the possibility they are present or if co-infection with insect only viruses is contributing to our observed differences in vector competence. The role of insect only viruses in vector competence is currently unresolved in *Ae. aegypti*, however *Cx. pipiens* infected with Culex flavivirus had a significantly higher proportion of mosquitoes develop a disseminated infection with WNV 7 days post infection (DPI), but not 14 DPI [41].

Local adaptation between the virus and mosquito vector has been documented before and demonstrates the need to use a viral isolate circulating in the same geographic region when making assumptions about vector competence. Lambrechts et al. [34] demonstrated that differences in vector competence among three *Ae. aegypti* collections from Thailand infected with three genotypes of DENV-1 was a result of mosquito genotype by virus genotype interactions. The current study builds on this and demonstrates mosquito genotype by virus genotype interactions also occur with sylvatic *Ae. aegypti* and with YFV.

Lambrechts et al. [34] and Bosio et al. [25] showed that the amount of virus in the midgut did not correlate with proportion of disseminated infections or the amount of virus outside the midgut. We found a slight correlation between the efficiency of viral replication in the midgut and the proportion of DIs. But this correlation was dependent on the virus. It is possible that the different viruses interact differently with the mosquito innate immune response, or that different genes in the mosquito are involved in the immune response to YFV or DENV-2. The use of plaque assays in the current study to quantify virus may result in different results than quantitative RT-PCR used by Lambrechts et al. [34] or TCID_50_ [25].

An advantage of the current study is that we were able to quantify the amount of virus in the midgut and disseminated infections through the use of plaque assays while other studies have only compared infection rates. Looking at quantitative differences in viral titers creates a more complete picture about how different viruses are interacting with different mosquito collections. The lack of variation in titers in the midgut compared to more variation in other tissues observed here could provide interesting insights into vector competence in different tissues. These results could point to different genes or different mechanisms in the mosquito being involved in viral defense inside the midgut as compared with the tissues outside the midgut. The mechanisms underlying *Ae. aegypti*/virus interactions remain unclear, but these results suggest virus specific mechanisms. Examining more collections throughout Senegal as well as infecting these collections with a non-sylvatic isolate of DENV-2 from Senegal may provide more insight into the mechanisms. Genetic association studies with different collections, different viruses, and different tissues might provide clues as to whether different genes in *Ae. aegypti* are important for a generalized or specific response to flaviviruses.

## Supporting Information

Table S1 Proportions of *Aedes aegypti* with DENV-2-JAM1409 infected midguts (MI) or disseminated infections (DI). Disseminated infections were assessed in head/thorax. Infection status was determined by plaque assay 14 days post infection.
(XLSX)

## References

[1] Bhatt S, Gething PW, Brady OJ, Messina JP, Farlow AW, et al (2013) The global distribution and burden of dengue. Nature 496: 504–507. 23563266 10.1038/nature12060 PMC3651993

[2] Gething PW, Kirui VC, Alegana VA, Okiro EA, Noor AM, et al (2010) Estimating the Number of Paediatric Fevers Associated with Malaria Infection Presenting to Africa's Public Health Sector in 2007. Plos Medicine 7: e1000301 doi:10.1371/journal.pmed.1000301 20625548 10.1371/journal.pmed.1000301 PMC2897768

[3] Mutebi JP, Barrett AD (2002) The epidemiology of yellow fever in Africa. Microbes and infection/Institut Pasteur 4: 1459–1468. 10.1016/s1286-4579(02)00028-x 12475636

[4] Organization TWH (2014) Yellow fever.

[5] Vasilakis N, Shell EJ, Fokam EB, Mason PW, Hanley KA, et al (2007) Potential of ancestral sylvatic dengue-2 viruses to re-emerge. Virology 358: 402–412. 17014880 10.1016/j.virol.2006.08.049 PMC3608925

[6] Vasilakis N, Cardosa J, Hanley KA, Holmes EC, Weaver SC (2011) Fever from the forest: prospects for the continued emergence of sylvatic dengue virus and its impact on public health. Nature reviews Microbiology 9: 532–541. 21666708 10.1038/nrmicro2595 PMC3321645

[7] Moncayo AC, Fernandez Z, Ortiz D, Diallo M, Sall A, et al (2004) Dengue emergence and adaptation to peridomestic mosquitoes. Emerging infectious diseases 10: 1790–1796. 15504265 10.3201/eid1010.030846 PMC3323252

[8] Zeller HG, Traore-Lamizana M, Monlun E, Hervy JP, Mondo M, et al (1992) Dengue-2 virus isolation from humans during an epizootic in southeastern Senegal in November, 1990. Research in virology 143: 101–102. 1594790 10.1016/s0923-2516(06)80088-9

[9] Saluzzo JF, Cornet M, Adam C, Eyraud M, Digoutte JP (1986) [Dengue 2 in eastern Senegal: serologic survey in simian and human populations. 1974–85]. Bulletin de la Societe de pathologie exotique et de ses filiales 79: 313–322. 3769119

[10] Cornet M, Saluzzo JF, Hervy JP, Digoutte JP, Germain M, et al (1984) Dengue 2 au Senegal oriental: Une poussee epizootique en milieu selvatique; isolements du virus a partir des moustiques et d′un singe et considerations epidemiologiques. Cah ORSTOM ser Ent Med et Parasitol 22: 313–323.

[11] Diallo M, Ba Y, Sall AA, Diop OM, Ndione JA, et al (2003) Amplification of the sylvatic cycle of dengue virus type 2, Senegal, 1999–2000: entomologic findings and epidemiologic considerations. Emerging infectious diseases 9: 362–367. 12643833 10.3201/eid0903.020219 PMC2958533

[12] Rico-Hesse R (1990) Molecular evolution and distribution of dengue viruses type 1 and 2 in nature. Virology 174: 479–493. 2129562 10.1016/0042-6822(90)90102-w

[13] Weaver SC, Vasilakis N (2009) Molecular evolution of dengue viruses: contributions of phylogenetics to understanding the history and epidemiology of the preeminent arboviral disease. Infection, genetics and evolution : journal of molecular epidemiology and evolutionary genetics in infectious diseases 9: 523–540. 19460319 10.1016/j.meegid.2009.02.003 PMC3609037

[14] Mutebi JP, Wang H, Li L, Bryant JE, Barrett AD (2001) Phylogenetic and evolutionary relationships among yellow fever virus isolates in Africa. Journal of virology 75: 6999–7008. 11435580 10.1128/JVI.75.15.6999-7008.2001 PMC114428

[15] McClelland GAH (1974) A worldwide survery of variation in scale pattern of the abdominal tergum of *Aedes aegypti* (L.) (Diptera: Culicidae). The Transactions of the Royal Entomological Society of London 126: 239–259.

[16] Mattingly PF (1958) Genetical aspects of the *Aedes aegypti* problem, I. Taxonomy and bionomics. Annals of Tropical Medicine and Parasitology 51: 392–408. 13498658

[17] Van Someren E.C.C HRB, Furlong M (1958) Observations on the behaviour of some mosquitoes of the Kenya coast. Bull ent Res 49: 643–660.

[18] Van Someren E.C.C. TC, Furlong M (1955) The mosquitoes of the Kenya coast; records of occurence, behaviour and habitat. Bull ent Res 46: 463–493.

[19] Mattingly PF (1958) Genetical aspects of the *Aedes aegypti* problem. II. Disease relationships, genetics and control. Annals of Tropical Medicine and Parasitology 52: 5–17. 13521698

[20] Brown JE, McBride CS, Johnson P, Ritchie S, Paupy C, et al (2011) Worldwide patterns of genetic differentiation imply multiple ‘domestications’ of Aedes aegypti, a major vector of human diseases. Proceedings Biological sciences/The Royal Society 278: 2446–2454. 10.1098/rspb.2010.2469 PMC3125627 21227970

[21] Tabachnick WJ, Powell JR (1979) A world-wide survey of genetic variation in the yellow fever mosquito, Aedes aegypti. Genetical research 34: 215–229. 544311 10.1017/s0016672300019467

[22] Sylla M, Bosio C, Urdaneta-Marquez L, Ndiaye M, Black WCt (2009) Gene flow, subspecies composition, and dengue virus-2 susceptibility among Aedes aegypti collections in Senegal. PLoS Negl Trop Dis 3: e408. 19365540 10.1371/journal.pntd.0000408 PMC2663788

[23] Huber K, Ba Y, Dia I, Mathiot C, Sall AA, et al (2008) Aedes aegypti in Senegal: genetic diversity and genetic structure of domestic and sylvatic populations. Am J Trop Med Hyg 79: 218–229. 18689628

[24] Tabachnick WJ, Wallis GP, Aitken TH, Miller BR, Amato GD, et al (1985) Oral infection of Aedes aegypti with yellow fever virus: geographic variation and genetic considerations. The American journal of tropical medicine and hygiene 34: 1219–1224. 3834804 10.4269/ajtmh.1985.34.1219

[25] Bosio CF, Beaty BJ, Black WCt (1998) Quantitative genetics of vector competence for dengue-2 virus in Aedes aegypti. Am J Trop Med Hyg 59: 965–970. 9886207 10.4269/ajtmh.1998.59.965

[26] Miller BR, Mitchell CJ (1991) Genetic selection of a flavivirus-refractory strain of the yellow fever mosquito Aedes aegypti. Am J Trop Med Hyg 45: 399–407. 1659238 10.4269/ajtmh.1991.45.399

[27] Diallo M, Ba Y, Faye O, Soumare ML, Dia I, et al (2008) Vector competence of Aedes aegypti populations from Senegal for sylvatic and epidemic dengue 2 virus isolated in West Africa. Transactions of the Royal Society of Tropical Medicine and Hygiene 102: 493–498. 18378270 10.1016/j.trstmh.2008.02.010

[28] Diallo M, Sall AA, Moncayo AC, Ba Y, Fernandez Z, et al (2005) Potential role of sylvatic and domestic African mosquito species in dengue emergence. The American journal of tropical medicine and hygiene 73: 445–449. 16103619

[29] Bennett KE, Olson KE, Munoz Mde L, Fernandez-Salas I, Farfan-Ale JA, et al (2002) Variation in vector competence for dengue 2 virus among 24 collections of Aedes aegypti from Mexico and the United States. Am J Trop Med Hyg 67: 85–92. 12363070 10.4269/ajtmh.2002.67.85

[30] Gubler DJ, Nalim S, Tan R, Saipan H, Sulianti Saroso J (1979) Variation in susceptibility to oral infection with dengue viruses among geographic strains of Aedes aegypti. The American journal of tropical medicine and hygiene 28: 1045–1052. 507282 10.4269/ajtmh.1979.28.1045

[31] Tardieux I, Poupel O, Lapchin L, Rodhain F (1990) Variation among strains of Aedes aegypti in susceptibility to oral infection with dengue virus type 2. The American journal of tropical medicine and hygiene 43: 308–313. 2221225 10.4269/ajtmh.1990.43.308

[32] Vazeille-Falcoz M, Mousson L, Rodhain F, Chungue E, Failloux AB (1999) Variation in oral susceptibility to dengue type 2 virus of populations of Aedes aegypti from the islands of Tahiti and Moorea, French Polynesia. The American journal of tropical medicine and hygiene 60: 292–299. 10072154 10.4269/ajtmh.1999.60.292

[33] van den Hurk AF, McElroy K, Pyke AT, McGee CE, Hall-Mendelin S, et al (2011) Vector competence of Australian mosquitoes for yellow fever virus. The American journal of tropical medicine and hygiene 85: 446–451. 21896802 10.4269/ajtmh.2011.11-0061 PMC3163864

[34] Lambrechts L, Chevillon C, Albright RG, Thaisomboonsuk B, Richardson JH, et al (2009) Genetic specificity and potential for local adaptation between dengue viruses and mosquito vectors. BMC evolutionary biology 9: 160. 19589156 10.1186/1471-2148-9-160 PMC2714696

[35] Sylla M, Ndiaye M, Black WC (2013) Aedes species in treeholes and fruit husks between dry and wet seasons in southeastern Senegal. Journal of Vector Ecology 38: 237–244. 24581351 10.1111/j.1948-7134.2013.12036.x

[36] Miller BR, Monath TP, Tabachnick WJ, Ezike VI (1989) Epidemic yellow fever caused by an incompetent mosquito vector. Tropical medicine and parasitology : official organ of Deutsche Tropenmedizinische Gesellschaft and of Deutsche Gesellschaft fur Technische Zusammenarbeit 40: 396–399. 2623418

[37] Sanchez-Vargas I, Scott JC, Poole-Smith BK, Franz AW, Barbosa-Solomieu V, et al (2009) Dengue virus type 2 infections of Aedes aegypti are modulated by the mosquito's RNA interference pathway. PLoS pathogens 5: e1000299. 19214215 10.1371/journal.ppat.1000299 PMC2633610

[38] Lunn DJ, Thomas A, Best N, Spiegelhalter D (2000) WinBUGS - A Bayesian modelling framework: Concepts, structure, and extensibility. Stat Comput 10: 325–337.

[39] McCarthy MA (2012) Bayesian Methods for Ecology. New York: Cambridge University Press.

[40] Miller BR, Mitchell CJ (1986) Passage of yellow fever virus: its effect on infection and transmission rates in Aedes aegypti. Am J Trop Med Hyg 35: 1302–1309. 3789277 10.4269/ajtmh.1986.35.1302

[41] Bolling BG, Olea-Popelka FJ, Eisen L, Moore CG, Blair CD (2012) Transmission dynamics of an insect-specific flavivirus in a naturally infected Culex pipiens laboratory colony and effects of co-infection on vector competence for West Nile virus. Virology 427: 90–97. 22425062 10.1016/j.virol.2012.02.016 PMC3329802

